# Long-lasting somatosensory cortex dysfunction in some, but not all, individuals after sport-related concussion

**DOI:** 10.1093/braincomms/fcag423

**Published:** 2026-11-04

**Authors:** Richard Wennberg, Audrey Potts, Youssef El-Rahimy, Dominik Zumsteg, Maria Carmela Tartaglia, Charles H Tator, Paul Comper, Mark Bayley, Luis Garcia Dominguez

**Affiliations:** Mitchell Goldhar Magnetoencephalography Unit, Toronto Western Hospital, Krembil Brain Institute, University Health Network, University of Toronto, Toronto, Ontario M5T 2S8, Canada; Canadian Concussion Centre, Toronto Western Hospital, Krembil Brain Institute, Toronto, Ontario M5T 2S8, Canada; Division of Neurology, Toronto Western Hospital, 399 Bathurst Street, Toronto, Ontario M5T 2S8, Canada; Mitchell Goldhar Magnetoencephalography Unit, Toronto Western Hospital, Krembil Brain Institute, University Health Network, University of Toronto, Toronto, Ontario M5T 2S8, Canada; Mitchell Goldhar Magnetoencephalography Unit, Toronto Western Hospital, Krembil Brain Institute, University Health Network, University of Toronto, Toronto, Ontario M5T 2S8, Canada; Klinik für Neurologie, Klinik Hirslanden, 8032 Zürich, Switzerland; Canadian Concussion Centre, Toronto Western Hospital, Krembil Brain Institute, Toronto, Ontario M5T 2S8, Canada; Division of Neurology, Toronto Western Hospital, 399 Bathurst Street, Toronto, Ontario M5T 2S8, Canada; Canadian Concussion Centre, Toronto Western Hospital, Krembil Brain Institute, Toronto, Ontario M5T 2S8, Canada; Hull-Ellis Concussion Clinic, Toronto Rehabilitation Institute, University of Toronto, Toronto, Ontario M5G 2A2, Canada; Hull-Ellis Concussion Clinic, Toronto Rehabilitation Institute, University of Toronto, Toronto, Ontario M5G 2A2, Canada; Mitchell Goldhar Magnetoencephalography Unit, Toronto Western Hospital, Krembil Brain Institute, University Health Network, University of Toronto, Toronto, Ontario M5T 2S8, Canada

**Keywords:** magnetoencephalography (MEG), middle-latency, mild traumatic brain injury (mTBI), sensory evoked fields (SEFs), somatosensory evoked potentials (SEPs)

## Abstract

Systems-level neurophysiologic dysfunction has been documented after acute sport-related concussion in multiple studies over the past quarter century. However, little is known about focal intracortical function post-concussion. We aimed to leverage the high sensitivity of magnetoencephalography (MEG) for the detection of neural activity in specific cytoarchitectonic areas of somatosensory cortex to determine whether focal intracortical changes in function could be identified after sport-related concussion. In a prospective case-control study short-latency and middle-latency sensory evoked fields (SEFs) evoked by median nerve stimulation at the wrist were compared in patients after acute sport-related concussion (*n* = 21; 9 female, age 29.0 ± 8.1 years) and non-injured controls (*n* = 26; 10 female, age 31.5 ± 11.3 years). The primary outcome measure was mean peak latency of the M60 (60 ms) middle-latency SEF. Secondary outcomes included M20, M30, M100 SEF latencies and correlation with clinical symptom scores. Follow-up MEG recordings were acquired 1, 2 and 4 months post-injury. At the group level, mean M60 latency was significantly delayed in patients compared with controls (77.07 ± 17.4 ms versus 60.86 ± 7.5 ms; *P* = 0.0001). Delays in M30 and M100 latencies (*p*_adj_ = 0.027 and 0.068, respectively) were also identified in the patient group. There was no difference in mean M20 latency between patients and controls (*p*_adj_ = 0.96). SEF latencies and clinical symptom scores were not correlated acutely or in follow-up. Symptoms improved significantly by 1-month follow-up with no significant improvements in SEF latencies. At the individual level a minority of patients (9/21) had a peak M60 latency delay >3 standard deviations above the control mean. Some affected patients demonstrated an unusual temporal dispersion of the M60 topographic field; follow-up recordings suggested that a very slow ‘reassembly’ of the M60 waveform can occur over time. Delays in SEF latencies were identified in a subset of patients in this study that affected all evoked responses after the initial M20 SEF, localizing the post-concussion deficit to intracortical grey matter and U-fibres, with functional sparing of thalamocortical white matter tracts. The altered SEFs represent focal dysfunction at the earliest stages of cortical processing in primary (Brodmann area 3b) and then secondary (Brodmann area 43) somatosensory cortex. The focal cortical dysfunction showed no correlation with clinical symptom scores, mirroring the systems-level dissociation between post-concussion clinical and neurophysiologic findings. At the individual level, only some patients exhibited abnormalities in early somatosensory processing, suggesting substantial inter-individual differences in susceptibility to neurophysiologic dysfunction post-concussion that will need to be accounted for in future studies.

## Teaser

Wennberg *et al*. report that magnetoencephalography detects delayed middle-latency sensory evoked fields after sport-related concussion, indicating focal intracortical dysfunction in primary and secondary somatosensory cortex with preserved thalamocortical pathways; abnormalities persist despite clinical recovery, vary across individuals, lack symptom correlation, and may slowly normalize over months to years.

## Graphical Abstract

**Graphical Abstract:**
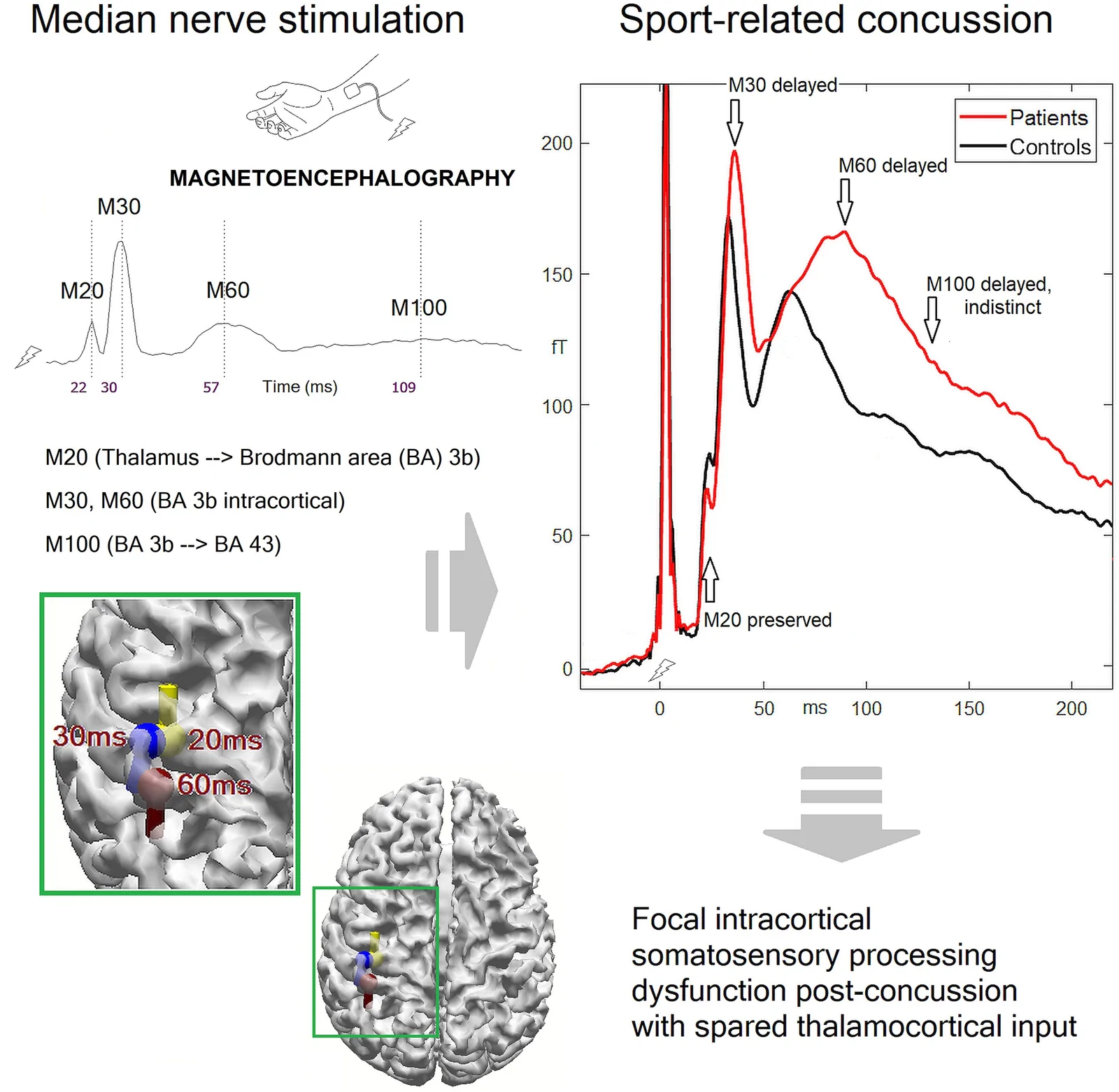

## Introduction

Little is known about focal intracortical changes in function that may occur in the days and weeks following sport-related concussion. Standard CT and MRI reveal no structural alterations and the main neurophysiologic evidence of cortical dysfunction comes from event-related potential (ERP) studies, which measure diffusely distributed cortical activity normally dependent on conscious attention.^1-11^

Unlike the distributed network ERPs recorded by EEG or magnetoencephalography (MEG), which occur with latencies between 100 and 500 ms after an external stimulus (usually a visual or auditory ‘oddball task’), earlier short-latency (20–40 ms) and middle-latency (40–100 ms) evoked responses can be recorded that are known to be focally generated in discrete cytoarchitectonic areas of primary cortex. These earlier responses are independent of conscious attention. In this context, the cortical responses evoked by electrical stimulation of the median nerve at the wrist are most commonly studied, and are particularly well-suited to MEG investigation owing to the tangential current flow orientation of the main components, which are generated in somatosensory cortex within the Rolandic and Sylvian fissures.^12,13^ The sequential series of short-latency and middle-latency responses, referred to as somatosensory evoked potentials (SEPs) if recorded by EEG and sensory evoked fields (SEFs) if recorded by MEG, represent the non-conscious initial processing and intracortical propagation of sensory input, before activation of downstream regions of association cortex.^14-18^

The first series of responses evoked in primary somatosensory cortex (S1) by median nerve stimulation at the wrist occur at ∼20 ms (peak I, generated in Brodmann area 3b), 25 ms (peak II, generated in Brodmann area 1), 30 ms (peak III, generated in Brodmann area 3b), 40 ms (peak IV, generated in Brodmann area 1), 50 ms (peak V, generated in Brodmann area 1 and/or 3a) and 60 ms (peak VI, generated in Brodmann area 3b). Peaks II and IV (from Brodmann area 1) are generally not separable as distinct waveforms in EEG, but can be better appreciated in voltage topographic maps (Fig. 1).^12,14^ The localization of Brodmann area 1 on the crown of the postcentral gyrus, and the resulting radial orientation of the dipolar field of electrical activation, typically renders MEG insensitive to the evoked responses around 25 and 40 ms, although extension of Brodmann area 1 into the central sulcus may create a tangential dipolar field of sufficient strength for MEG detection, as can be seen in the SEFs obtained in response to left-sided stimulation in control subject 1 (Supplementary Fig. 1). The middle-latency peaks V and VI are most evident in EEG and MEG, respectively, and are compatible with local intracortical propagation of activation within S1 from a predominantly radial SEP orientation (at peak V) to a predominantly tangential SEF orientation (at peak VI). A later middle-latency response at around 100 ms (peak VII), not uniformly identifiable in EEG or MEG recordings, is generated within the upper bank of the Sylvian fissure in secondary somatosensory cortex (S2; Brodmann area 43) as a result of direct cortico-cortical activation via U-fibres from S1 (Fig. 1, Supplementary Figs 1 and 2).^15,19^ The main short-latency and middle-latency peaks of the SEFs reliably recorded with MEG occur at I (M20), III (M30), VI (M60), and, less consistently, VII (M100), the first three generated in S1 (Supplementary Fig. 3) and the last in S2.^13^

**Figure 1:**
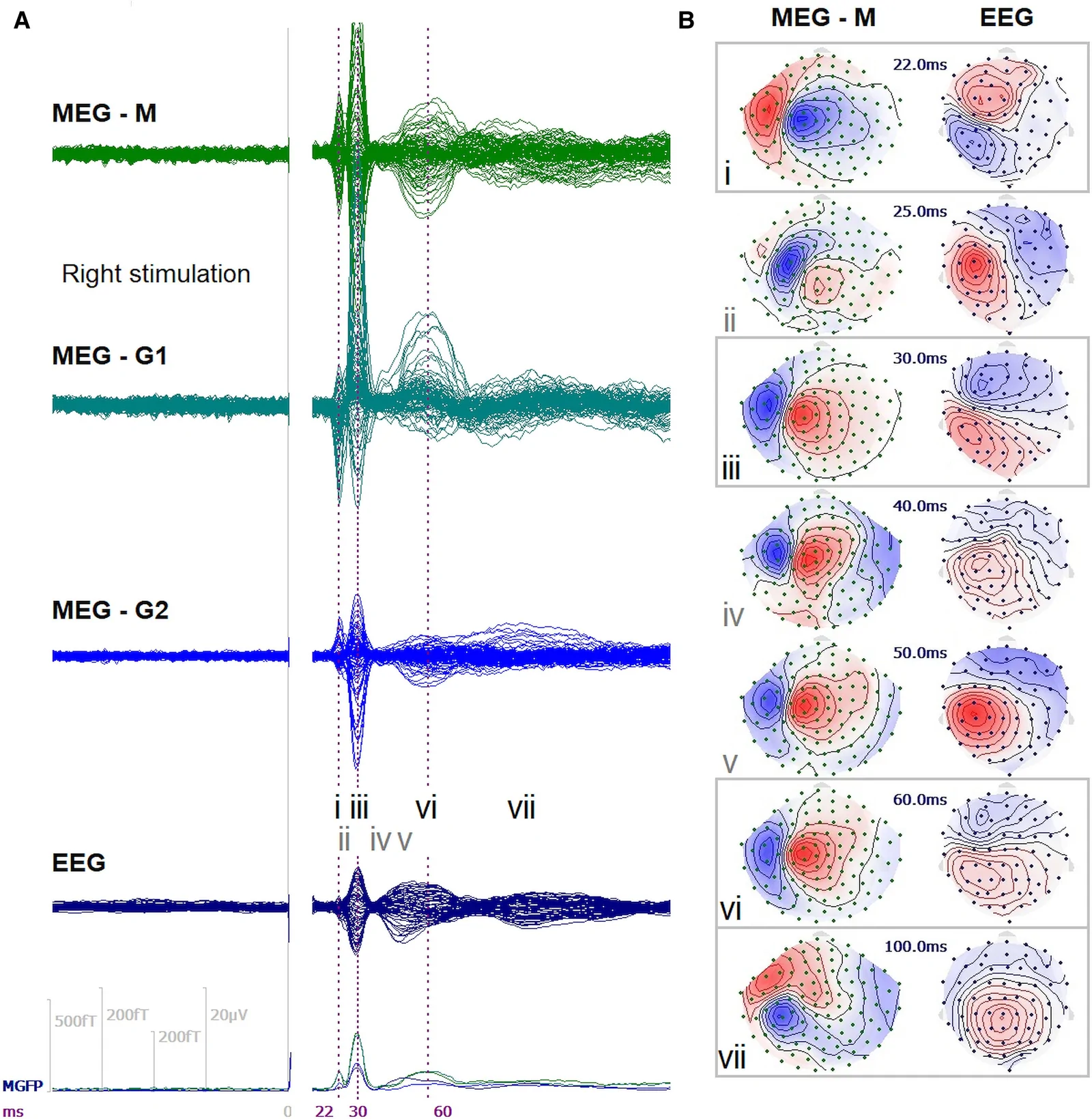
**Normal somatosensory cortex responses to median nerve stimulation at the wrist.** Control subject 1, simultaneous MEG-EEG recording. Right wrist stimulation. (**A**) EEG and MEG evoked responses in primary somatosensory cortex (S1) occur at approximately 20 ms (I), 25 ms (II), 30 ms (III), 40 ms (IV), 50 ms (V) and 60 ms (VI); for MEG, the responses at I (M20), III (M30) and VI (M60) are the main sensory evoked fields (SEFs). The less distinct, lower amplitude evoked response around 100 ms (VII) is generated in secondary somatosensory cortex (S2). More details in text and Supplementary Fig. 2. Electromagnetic stimulation artefact (at *t* = 0) removed from figures for clarity. (**B**) Topographic plots show the corresponding MEG magnetometer flux (red = flux out of head, blue = flux into head) and EEG voltage (red = electropositive, blue = electronegative) fields at peaks I-VII. Rectangles highlight the field topographies at the main MEG peaks; by the ‘right hand rule’ of electromagnetism the magnetic flux fields are oriented at 90° to the corresponding voltage fields (see also Supplementary Fig. 2). M = magnetometers. G1 = planar gradiometers. G2 = planar gradiometers (orthogonal to G1). MGFP = mean global field power.

Short-latency SEPs have not been found useful for the detection of focal intracortical post-concussion changes^3,17^; however, evidence from a previous study has suggested that middle-latency SEPs might serve as a biomarker of both cortical dysfunction post-concussion and of subsequent recovery.^17^ In this work, we aimed to leverage the high sensitivity of MEG for the tangentially-oriented peri-Rolandic and peri-Sylvian dipolar fields of middle-latency median nerve SEFs to further explore the possibility that focal changes in somatosensory cortex function may occur post-concussion. Specifically stated, we hypothesized that sport-related concussion impairs somatosensory cortex function, manifesting as delayed M60 SEF latencies.

## Materials and methods

### Study design

This was a prospective case-control study of the effects of acute concussion (independent variable) on middle-latency SEFs (dependent variables). The primary outcome measure was mean peak latency of the M60 waveform; secondary outcome measures included peak latencies of the M20, M30 and M100 SEFs, changes in mean latencies at 1-, 2- and 4-month follow-up recordings, and correlation with total symptom severity scores acutely and in follow-up. Symptom scores were obtained using the Sport Concussion Assessment Tool 5th edition (SCAT5).^20^

Assuming the same large effect size (*d* = 0.884) seen for delayed middle-latency SEPs in a pilot study of post-concussion patients,^17^ to detect with 80% power a significant (*P* < 0.05, one-tailed) group level difference in means of the primary outcome measure we calculated that a sample size of 36 subjects (18 controls, 18 patients) would be required.

### Participants

Control subjects (*n* = 26, 10 female, mean age 31.5 ± 11.3 years, range 15–55) were volunteers recruited from among neurophysiology technologists, research associates, family members, neurologists and neurology residents affiliated with the University Health Network clinical neurophysiology laboratory. Control subjects had no history of post-concussion syndrome or recent (within 2 years) head injury, and no other neurological condition.

Patients (*n* = 21, 9 female, mean age 29.0 ± 8.1 years, range 16–43) with acute sport-related concussion were consecutively recruited from the Hull-Ellis Concussion Clinic, Toronto Rehabilitation Institute (*n* = 17) and the Canadian Concussion Centre, Toronto Western Hospital (*n* = 4). Patients were assessed clinically within one week of injury. MEG recording was obtained within 10 days of acute concussion and again at 1-, 2- and 4-month follow-up. Weekly clinical follow-up, including SCAT5 completion, was obtained for patients at the Hull-Ellis Concussion Clinic. Neuroimaging was not routinely obtained; in a minority of cases brain CT or MRI scans were acquired either acutely or during the follow-up period, with no structural abnormalities identified. Patients had no past history of persisting concussion symptoms, and no other neurological condition; four (patients 3, 8, 10 and 12) had suffered previous sport-related concussions 2–17 years prior to study enrolment (Supplementary Table 1). Written informed consent was provided by patients and the study approved by the institutional Research Ethics Board.

### MEG recordings

Recordings were acquired using an Elekta Neuromag TRIUX 306-channel MEG system (Helsinki, Finland). Sampling frequency was 1000 Hz and online filter bandwidth 0.1–330 Hz. Artefact suppression used the default parameters (10-s time window, subspace correlation 0.980) of the spatiotemporal signal space separation algorithm implemented within the Elekta Maxfilter suite.^21,22^ No other preprocessing was performed, and data quality did not differ between patients and controls. Head position inside the sensor array was determined from five coils attached to the scalp; a Polhemus 3D-Fastrak system (Colchester, USA) was used to digitize the location of the head position indicator coils. For illustrative purposes only, simultaneous 64-channel EEG recording was acquired in one control subject to confirm and demonstrate the typical expected SEP/SEF electric and magnetic field orientations.

Stimulation was carried out using square-wave current pulses 0.2 ms in duration delivered in an alternating pseudorandomized fashion to the right and left median nerves at the wrist with an inter-stimulus interval between 1 and 2 s. The SEF epoch duration was 600 ms with trigger offset −100 ms. Stimulus intensity was adjusted slightly above motor threshold to produce a moderate thumb twitch. Recordings were obtained with subjects resting comfortably, recumbent with eyes closed; 200–500 stimuli were administered to each side.

### Sensory evoked field (SEF) latency measurements

Peak latencies of the M20, M30 and M100 SEFs were visually identified in butterfly plots of the 306-channel raw MEG data and associated mean global field power (MGFP) waveforms, interpreted side-by-side the whole-head flux field topographies to ensure accurate identification of each sequential evoked response.

M60 SEFs appeared unusually temporally dispersed in some patients on one or both sides (see Results), which rendered definitive peak identification in such cases difficult in the raw MEG or MGFP waveforms. To strengthen the signal-to-noise ratio (SNR) of the main contribution to the M60 SEF topography, principal component analysis (PCA) was performed across the entire M60 segment and the peak latency defined as the timepoint of maximal SNR of the first PCA component. For consistency, the same method was used for all M60 SEF peak determinations in patients and controls.

Given that the PCA method described above is novel, as a comparative validation the M60 responses were analyzed using a fractional (50%) area latency method. In this method, the timepoints of the onset and offset of the M60 topographic waveform were marked (using the magnetometer and corresponding MGFP data) and the time range under the M60 waveform divided into two equal halves, with the resulting timepoint identified as the ‘peak’. Analysis in this way does not identify a true peak, but may account for the dispersion of the M60 waveform in a way the PCA method cannot.

To further compare and contrast the spatiotemporal distributions of short-latency and middle-latency SEFs in acute concussion patients and controls, topographic dissimilarity (global map dissimilarity) maps were created using FieldTrip.^23^

For illustrative purposes only, magnetic source imaging was performed in two control subjects using Curry 6 (Compumedics, Abbotsford, Australia) and previously described methods^24,25^ to demonstrate the typical dipole source localization and orientation of the main SEF peaks in somatosensory (S1 and S2) cortex.

### Statistical analyses

Statistical between-group comparisons of SEF latencies (acute concussion patients versus controls) were performed using the non-parametric Mann-Whitney U-test, incorporating the two independent measurements (left and right stimulation) obtained per subject. The same between-group comparisons were also performed after averaging the bilateral latencies obtained per subject prior to non-parametric testing. For the primary outcome measure (M60) no adjustment for multiple comparisons was applied. For the secondary outcome measures (M20, M30 and M100) adjusted *P*-values (*p*_adj_) were calculated using the Holm-Bonferroni method.

Longitudinal patient follow-up analyses of SEF latencies were performed using the Mann-Whitney U-test with *P*-values adjusted using the Holm-Bonferroni method. Left and right median nerve SEFs (M20, M30, M60, M100) were treated as repeated measurements within subjects.

Spearman rank correlation was used to assess correlation between M60 SEF latency values and SCAT5 total symptom severity scores in patients at acute, 1-, 2- and 4-month time points, with *P*-values adjusted for multiple comparisons across time points using the Holm-Bonferroni method. The Mann-Whitney U-test was used for statistical comparison of group level acute and 1-month follow-up SCAT5 scores.

Statistical significance for all two-tailed tests was set at *α* = 0.05.

## Results

Individual patient demographics and results, including sport, SCAT5 scores and M30, M60 and M100 SEF latencies are presented in Supplementary Table 1. Technical difficulties prevented recording from one side in patient 18. M20, M30 and M60 peaks were identified in all other acute concussion patient recordings (*n* = 41 measurements); M100 peaks were identified in 31/41 acute recordings in 17/21 patients. Recordings were acquired in 16 patients (*n* = 31 measurements) at 1-month follow-up, 12 patients (*n* = 23 measurements) at 2-month follow-up, and 8 patients (*n* = 16 measurements) at 4-month follow-up. In patient 1, long-term follow-up recording was also available outside the study design, acquired 5 years post-concussion.

Demographics and SEF latencies of controls are presented in Supplementary Table 2. M20, M30 and M60 peaks were identified in all control subject recordings (*n* = 52 measurements); M100 peaks were identified in 43/52 recordings in 24/26 controls.

There was no difference in age between groups (Welch’s *t*-test, *t* = 0.8983, df = 44, standard error = 2.826, *P* = 0.3739).

At the group level, for the primary outcome measure, a highly significant delay in mean M60 peak latency was found when comparing acute concussion patients to controls (77.07 ± 17.4 ms versus 60.86 ± 7.5 ms; *U* = 569.5, *z* = −3.84, *P* = 0.0001). Figure 2A shows the grand averaged SEF waveforms of the two groups, where a slight delay of the M30 peak is also evident in the patient group, as is a loss of resolution of the M100 peak in the averaged waveform. Also apparent in the averaged waveforms is a higher mean amplitude of SEFs after the M20 response in the patient group (amplitude was not a pre-defined outcome measure and not examined further in this study). Figure 2B presents box plot comparisons of peak latencies of the four main SEFs in acute concussion patients and controls. In addition to the highly significant difference in the primary outcome (M60) measure, delays were found in the patient group compared with controls for the secondary outcome measures of M30 (34.95 ± 2.6 ms versus 33.38 ± 3.1 ms; *U* = 729.5, *z* = −2.60, *P* = 0.009, *p*_adj_ = 0.027) and M100 (128.97 ± 29.4 ms versus 114.14 ± 17.6 ms; *U* = 860, *z* = −2.12, *P* = 0.034, *p*_adj_ = 0.068) peak latencies. There was no difference between patients and controls in M20 SEF latencies (22.10 ± 1.1 ms versus 22.13 ± 1.6 ms; *U* = 1060, *z* = 0.05, *P* = 0.96, *p*_adj_ = 0.96).

**Figure 2:**
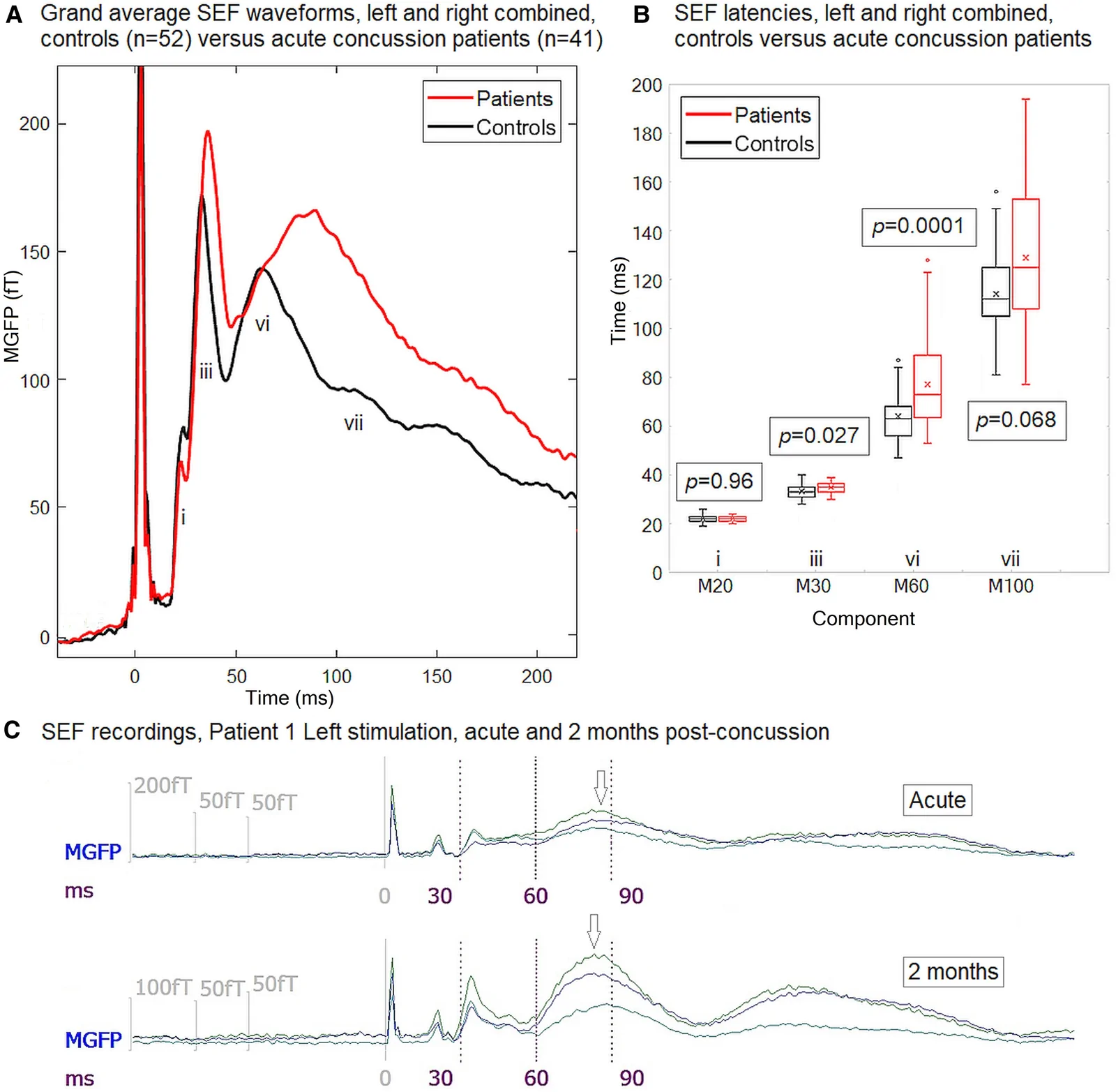
**Median nerve sensory evoked field (SEF) latencies, patients and controls.** (**A**) Grand average of SEF waveforms demonstrating increased latencies (and amplitudes) in acute patient group at peaks III and VI, and loss of resolution of peak VII. (**B**) Significant latency increases in acute patient group for SEF components III (M30; Mann-Whitney U-test, *U* = 729.5, *p*_adj_ = 0.027) and VI (M60; *U* = 569.5, *P* = 0.0001); non-significant increase for SEF component VII (M100; *U* = 860, *p*_adj_ = 0.068). No difference in M20 latency (peak I) between patients and controls (*U* = 1060, *p*_adj_ = 0.96). Number of data points: controls, 52 (M20, M30, M60), 43 (M100); patients, 41 (M20, M30, M60), 31 (M100). (**C**) Patient 1, left wrist stimulation, shows delayed peak VI latency (arrow) after acute concussion (*top*) with slight leftward correction (arrow) 2 months post-concussion (*bottom*). fT = femtotesla. MGFP = mean global field power.

Using the averaged bilateral latencies per subject to compare the acute concussion group (*n* = 21) with the control group (*n* = 26) at the subject (rather than individual measurement) level, a similar significant delay in mean M60 peak latency was found in the patient group (77.04 ± 16.2 ms versus 63.98 ± 8.9 ms; *U* = 142.5, *z* = 2.79, *P* = 0.005). Analyzed in this way (averaged bilateral latencies per subject), and after adjustment for multiple comparisons, there were no significant delays found in the patient group compared with controls for the secondary outcome measures of M20 (22.05 ± 1.13 ms versus 22.13 ± 1.5 ms; *U* = 265.5, *z* = −0.16, *P* = 0.87, *p*_adj_ = 0.87), M30 (35 ± 2.5 ms versus 33.38 ± 2.8 ms; *U* = 179, *z* = 2.01, *P* = 0.044, *p*_adj_ = 0.132) and M100 (128.85 ± 32.0 ms versus 114.10 ± 16.8 ms; *U* = 255.5, *z* = 1.36, *P* = 0.172, *p*_adj_ = 0.346) peak latencies.

Supplementary Table 3 shows the results obtained when analyzing the M60 waveform using the area fractional latency method; Supplementary Fig. 4 illustrates the technique. This method of analysis accentuated the between-group differences at the subject level (means = 82.71 ± 13.4 ms versus 66.85 ± 7.0 ms; *U* = 84, *z* = 4.04, *P* = 0.00005).

At the individual level, despite the highly significant group level difference in the primary outcome measure, only 9 of 21 acute concussion patients (in 15/41 recordings) had a peak M60 latency more than three standard deviations above the control mean. There was no correlation between M60 latencies and acute SCAT5 scores (*ρ* = −0.255, *P* = 0.64, *p*_adj_ = 1.0).

Some patients with delayed M60 peaks showed improvement (decreased latency) at follow-up recording (Fig. 2C); however, most did not. The SEF peak latencies showed no correlation with SCAT5 scores at 1-, 2- or 4-month follow-up (all *p*_adj_ ≥ 0.69).

As aforementioned in the Methods, an unexpected temporal dispersion of the M60 SEF was observed in some patients. An example of such a case in shown in Fig. 3, along with the PCA method used to identify the peak maximum of the evoked response. Another example is demonstrated in Supplementary Fig. 4. At the group level, this tendency toward an extension in time of the M60 topographic flux field in patients is demonstrated in the dissimilarity plot contrasting the SEF topographies of acute concussion patients versus controls (Fig. 4A).

**Figure 3:**
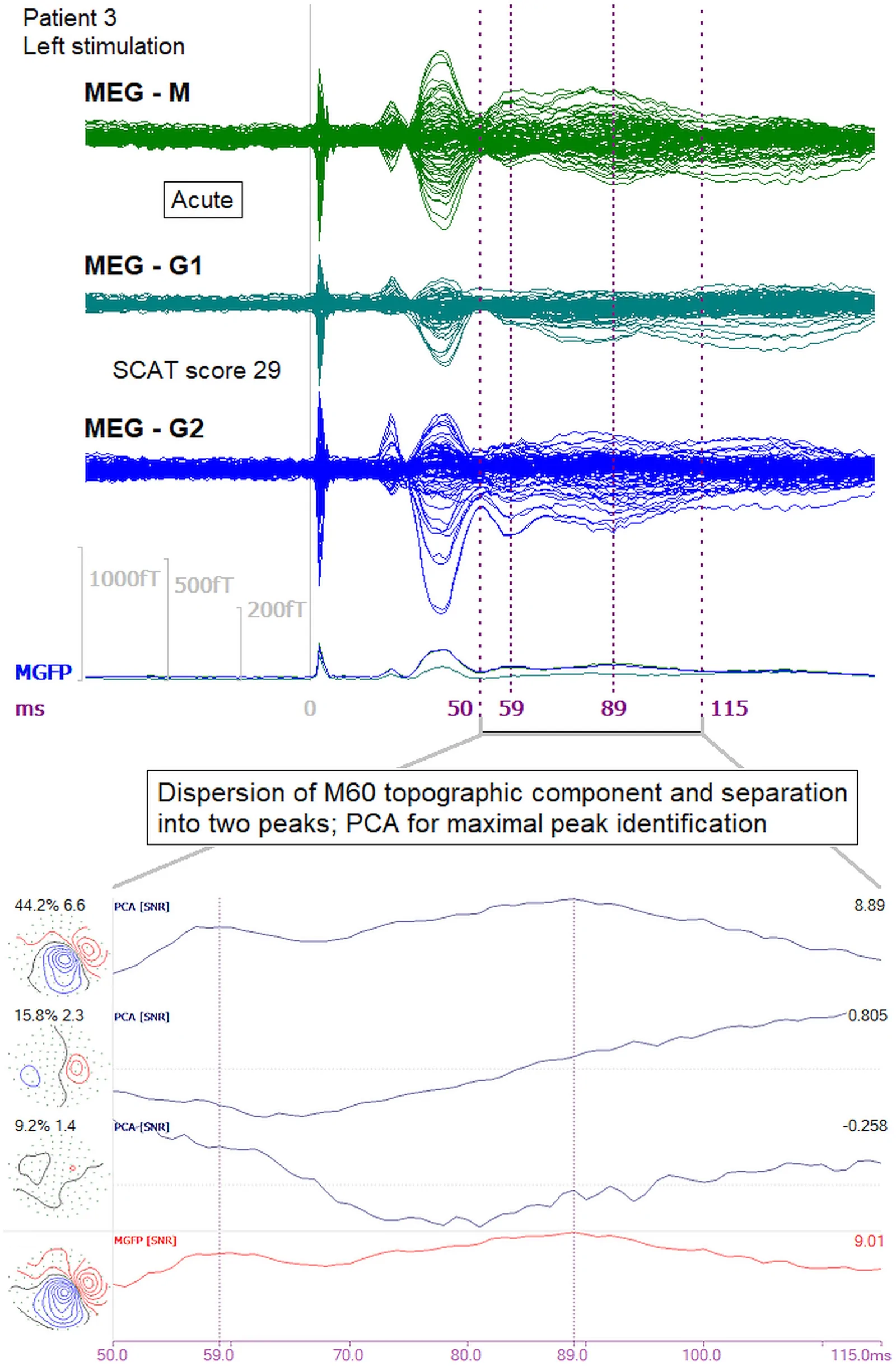
**Dispersion of M60 response acutely, PCA method for identification of M60 peak.** Patient 3, acute post-concussion recording, showing dispersion of topographic M60 component and separation into two main latency peaks around 59 and 89 ms (*top*). PCA of the M60 segment (*bottom*) reveals a peak maximum of the first PCA component at 89 ms. Upper three PCA rows show the first three extracted components; the bottom row shows the mean global field power (MGFP) of the non-decomposed signal. Numbers in the left column of each PCA row show the explained variance (*left*) and signal-to-noise ratio (SNR, *right*) of each extracted component in the selected time segment (50–115 ms, underlined). Numbers at right of each PCA row indicate SNR at timepoint marked by the vertical line at 89 ms. Topographic plots show the MEG magnetometer flux field corresponding to the PCA and MGFP data. fT = femtotesla.

**Figure 4:**
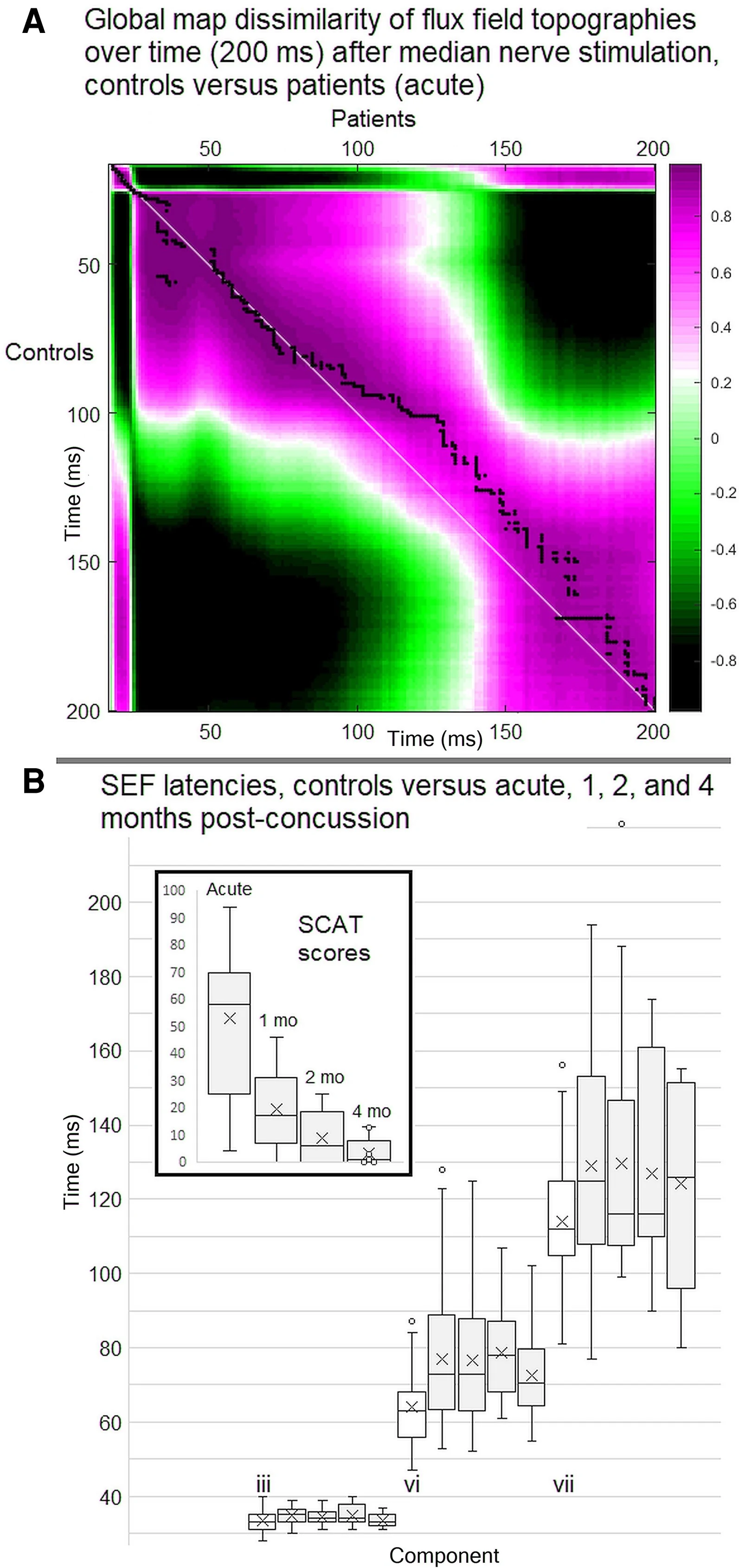
**Dispersion of M60 response in topographic dissimilarity map; symptom resolution and neurophysiologic recovery unrelated over first 4 months post-concussion.** (**A**) Dissimilarity map of flux field topographies shows a divergence from controls in the acute patient group maximal between 75 and 175 ms. Colour scale represents normalized topographic similarity, ranging from −1 (low) to +1 (high). Number of measurements: controls, 52; patients, 41. (**B**) Patient group decrease in total symptom severity scores at 1-, 2- and 4-months post-concussion (inset) is much more evident than improvements in abnormal SEF latencies at same time points. Group level improvement in SCAT scores significant at 1-month follow-up (Mann-Whitney U-test, *U* = 49.5, *P* = 0.001); no significant group level improvements in SEF latencies during follow-up (see text). Number of datapoints for SCAT scores: 19, 16, 10, 5 (acute, 1 mo, 2 mo, 4 mo, respectively). Number of datapoints for SEF peak latencies: controls, 52 (III, VI), 43 (VII); patients acute, 41 (III, VI), 31 (VII); patients 1 mo, 31 (III, VI), 25 (VII); patients 2 mo, 23 (III, VI), 19 (VII); patients 4 mo, 16 (III, VI), 13 (VII). Inside box X = mean; line = median. Components III, VI, VII = M30, M60, M100 SEF peaks, respectively. fT = femtotesla. SCAT = Sport Concussion Assessment Tool 5th edition.

In follow-up, group level analysis showed significant improvement in post-concussion SCAT5 scores at 1-month follow-up (*U* = 49.5, *z* = 3.23, *P* = 0.001), and further slight improvements at 2- and 4-month follow-up (Fig. 4B). In contrast, group level analysis of mean SEF peak latencies showed no significant improvements in M30, M60 or M100 peak latencies at 1-, 2- or 4-month post-concussion recording (all *p*_adj_ > 0.45; Fig. 4B).

The unusual temporal prolongation of the M60 SEF in some patients was explored in more detail during follow-up recording and it appeared that in some cases—even if the PCA-identified M60 peak latency was unchanged—a gradual ‘reassembly’ of the M60 waveform into a narrower, leftward-shifted evoked response was occurring over time, as shown for patients 15 and 16 in Fig. 5. In a singular case (patient 1) we were able to obtain follow-up MEG 5 years post-concussion and the recording showed a marked narrowing and leftward normalization of the M60 SEF (Fig. 6), providing some support for the ‘reassembly’ hypothesis hinted at in patients 15 and 16.

**Figure 5:**
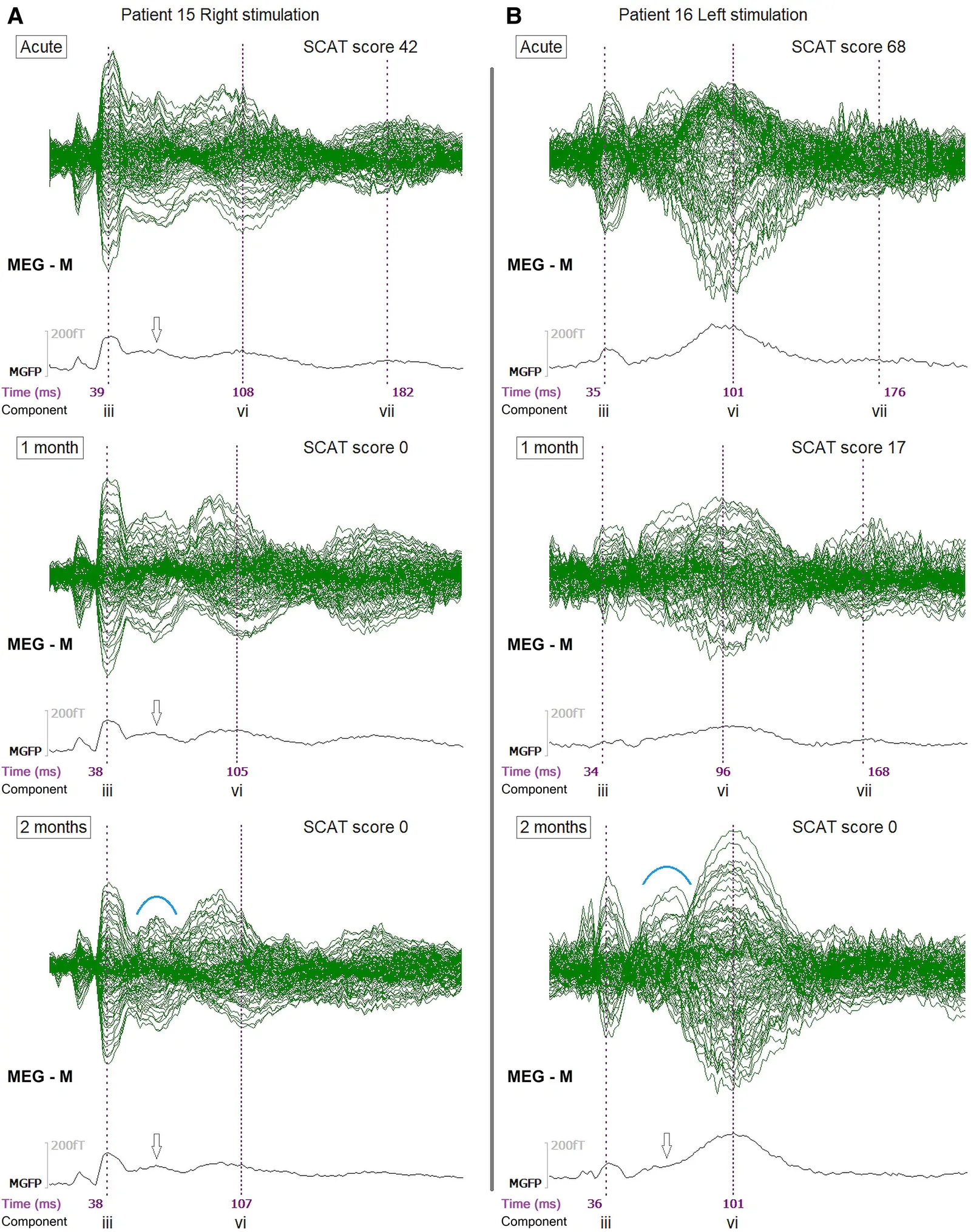
**Gradual reassembly of M60 response in some patients over months post-concussion.** (**A**) Patient 15, acute post-concussion recording (*top*) showing dispersion of M60 response with a delayed main peak (PCA first component) at 108 ms and a second, less well-defined peak in the normal M60 range (arrow). One month (*middle*) and 2 months (*bottom*) later, symptom-free, main M60 PCA-identified peak shows similar delayed latency but earlier peak (arrows, semicircle) appears increasingly well-defined. (**B**) Patient 16, acute (*top*) and 1 month (*middle*) post-concussion recordings showing delayed, dispersed M60 response. Two month (*bottom*) post-concussion recording, symptom-free, shows similar delay of main M60 peak but development of a more distinct earlier peak in the normal M60 range (arrow, semicircle). fT = femtotesla. M = magnetometers. MGFP = mean global field power. SCAT score = Sport Concussion Assessment Tool 5th edition total symptom severity score.

**Figure 6:**
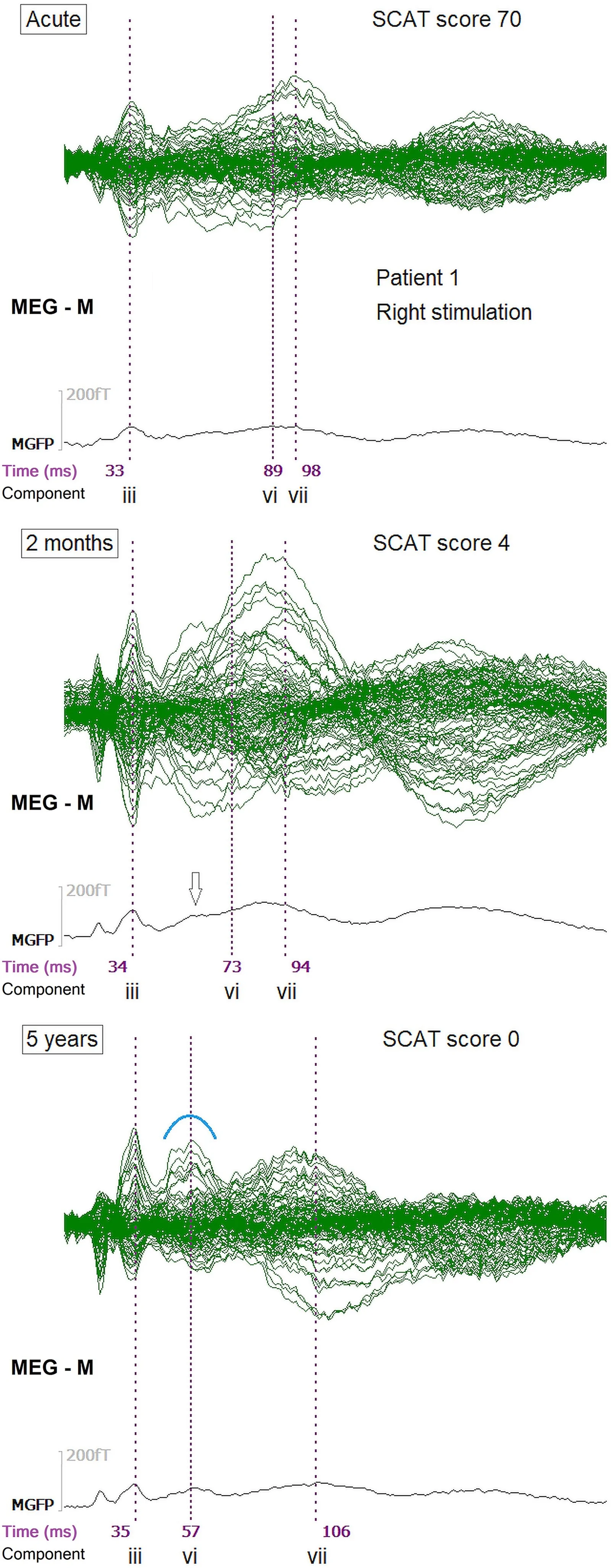
**Gradual reassembly of M60 response in one patient over months and years post-concussion.** Patient 1, acute post-concussion recording (*top*) showing delayed M60 response at 89 ms, which topographically overlapped with the patient’s normal PCA-identified M100 peak at 98 ms. After 2 months (*middle*), markedly improved clinically but not symptom-free, main PCA-identified M60 peak has shifted leftward to 73 ms, with the appearance of a second, earlier peak (arrow). Follow-up recording obtained in this patient 5 years post-concussion (*bottom*), symptom-free, shows normalization of the M60 response with a single peak at 57 ms. fT = femtotesla. M = magnetometers. MGFP = mean global field power. SCAT score = Sport Concussion Assessment Tool 5th edition total symptom severity score.

One control subject (control 25, Supplementary Table 2) suffered a sport-related concussion during the study period, and we were able to acquire acute post-concussion MEG in the same individual (the participant was redesignated as patient 4 post-injury, Supplementary Table 1). In this case, the post-concussion recording showed no changes in SEF latencies or topographies compared with the pre-injury recording, despite the presence of clinical symptoms (Supplementary Fig. 5).

## Discussion

Neurophysiologic evidence of diffuse cortical dysfunction post-concussion has been identified, at least at the group level, in many ERP or functional connectivity studies over the past quarter century.^1,2,4-11,26,27^ These systems-level analyses of conscious cortical network interactions do not, however, provide any insight into focal intracortical changes in function that may result from acute concussion.

MEG is theoretically well-positioned to reveal focal dynamic changes in cortical activity, not only because of its millisecond temporal resolution but also due to its high spatial resolution, especially for tangentially-oriented dipolar fields generated within the large fissures on the lateral convexity of the brain.^13,25^ The prototypical technique for evoking such focal cortical activity is electrical stimulation of the median nerve at the wrist.

Our study of median nerve SEFs, comparing acute sport-related concussion patients with non-injured controls, identified a clear difference in the primary outcome measure (M60 latency) at the group level. Moreover, the mean latencies of all SEFs that follow sequentially the arrival of the first cortical input from the periphery to S1 (marked by the M20 SEF) were delayed at the group level, including the short-latency M30. These delayed SEFs in the post-concussion group represent focal dysfunction at the earliest stages of intracortical somatosensory processing, first in S1 and then S2.

The finding that the M20 latency remains intact while the subsequent M30 and M60 latencies are delayed is an important observation that localizes the post-concussion deficit to intracortical grey matter signal propagation (in Brodmann area 3b) and U-fibres, with functional sparing of thalamocortical white matter tracts. Although the precise synaptic, cellular or molecular alterations underlying the intracortical and U-fibre dysfunction are unclear they appear to affect both the speed and efficiency of cortical sensory processing, as manifest in the delayed peak latencies and temporal dispersion of the M60 SEFs, respectively.

The significant differences identified at the group level were driven by a minority of subjects in whom large changes in focal S1 and/or S2 function had occurred post-concussion. Still, 43% of acute concussion patients showed unequivocal abnormalities in early cortical sensory processing detectable by MEG. For practical purposes, this implies that the measurement of SEFs using MEG cannot be envisioned as a sensitive test to determine whether or not an individual has sustained an acute concussion. At the pathophysiological level, however, the observation that some, but not all, patients showed post-concussion alterations in somatosensory cortex function is a novel indication that individual differences in neurophysiologic susceptibility to concussion may be profound. The focal abnormalities identified in affected patients are clearly neuropathological, unrelated to psychological or attentional factors, given that short-latency and middle-latency SEFs are not dependent on conscious attention. Nonetheless, the abnormalities showed no correlation with clinical symptom severity scores, neither acutely nor in follow-up.

The observation that abnormal post-concussion SEF findings were not correlated with SCAT5 scores is compatible with many previous ERP studies showing a dissociation between subjectively reported clinical symptoms and objective neurophysiologic changes, and especially with a persistence of neurophysiologic abnormalities after clinical symptom resolution.^1,4,6,7,28^ But the dissociation in this SEF study shows in addition something slightly different from the ERP studies, which is that in some individuals prominent post-concussion symptoms may be present in the absence of focal non-conscious somatosensory cortex dysfunction. One must presume that the more diffuse association cortex dysfunction detectable in ERP studies may in some cases be strictly limited to changes in functional network connectivity between distant brain regions,^11^ with no involvement of somatosensory cortex. Notwithstanding, such systems-level dysfunction could, or perhaps must, be related to underlying focal intracortical dysfunction affecting gyri somewhere other than somatosensory cortex. Unfortunately, techniques to non-invasively assess focal neurophysiologic function with high fidelity in non-somatosensory cortex are limited.

As with the above-mentioned dissociations between clinical recovery and persistent ERP deficits, it is notable that the focal deficits in somatosensory cortex function detected by MEG can also be subclinical. Neither symptom severity nor age correlated with M60 SEF abnormalities, acutely or in follow-up (Supplementary Tables 1–3, Supplementary Fig. 6). Irrespective of age, the focal deficits are presumably compensated for by cognitive reserve in some individuals within weeks to months of injury.

There was no significant group level improvement in mean SEF latencies during the pre-defined follow-up period; however, attrition of the patient cohort at 2 and 4 months will have increased the risk of a Type II error, and improvements in SEF latencies over time were noted in some individuals. In particular, the observation of a very gradual normalization process, extending over months to years, of the temporally dispersed M60 SEF waveform recorded in some patients was an intriguing discovery, one which we have described as ‘reassembly’ in this paper.

Our use of the term ‘reassembly’ in this context is not meant to indicate any known cellular mechanism of repair, but simply an attempt to describe in one word the observed macroscopic narrowing of the M60 flux field over time. We presume the narrowing of the waveform is due to resynchronization of post-synaptic potential (PSP) input onto pyramidal cells in Brodmann area 3b, around the time of the M60 evoked response. The apparent separation of the M60 waveform into two identifiable peaks in some cases suggests that selective damage affecting preferentially the intracortical U-fibres, with relative sparing of the juxtacortical U-fibres (or vice versa), could be a neuropathological mechanism, with slow remyelination of the more damaged U-fibre track leading to the gradual resynchronization of PSPs. However, this is speculative. Elucidation of the underlying mechanism(s) of synaptic, cellular or molecular post-concussion repair in somatosensory cortex must await the results of future investigations.

The most important limitation of our study was the attrition of patients during follow-up, which resulted in the longitudinal portion of the study being underpowered to detect mild or moderate group level improvements in SEF latencies at final follow-up. A potential limitation was the temporal dispersion of the M60 waveform discovered in a subset of patients, which presented challenges for the reliable identification of single peak timepoints, and resulted in the need to resort to a novel PCA method for peak identification. However, our use of the alternate 50% area latency method returned similar results for group level differences between acute patients and controls, and the convergence of findings from these two very different methods is reassuring. The age range of subjects in our study was broad, and middle-latency evoked response latencies have been shown to increase with age. However, such age-related latency increases are noted at the population level only after the age of 50 years,^29^ and thus would not affect our patient cohort. There is a common conviction that a general limitation of middle-latency SEPs/SEFs is the existence of high variability even in healthy individuals;^29^ however, inter-individual variability would not affect the between-group comparisons of this study and intra-individual replicability appears high (see examples in Supplementary Figs 2A and 5). Finally, it would have been ideal to have high resolution brain MRI scans available for all patients and controls to enable magnetic source imaging in all participants, but this was not a part of the study design, which extended beyond routine clinical care only in the acquisition of MEG.

In conclusion, our findings demonstrate that middle-latency SEFs provide a novel, objective biomarker of focal intracortical dysfunction post-concussion, with the potential for tracking gradual neurophysiologic recovery at the individual level. The altered SEF latencies observed in some, but not all, individuals after sport-related concussion reveal focal somatosensory processing deficits that persist beyond clinical recovery, suggesting a neurophysiologic heterogeneity that will need to be accounted for in future studies.

## Supplementary Material

fcag423_Supplementary_Data

## Data Availability

Data will be made available from the corresponding author upon reasonable request from a qualified investigator.
