# Supplementary material for: Long-lasting somatosensory cortex dysfunction in some, but not all, individuals after sport-related concussion
Source: Brain Commun. 2026 Nov 4;8(6):fcag423. doi: 10.1093/braincomms/fcag423 (PMC13766682; doi:10.1093/braincomms/fcag423)
Supplement: Supplementary file 1 [file fcag423_supplementary_data.pdf]

Supplementary material for **Long-lasting somatosensory cortex dysfunction in some, but not all, individuals after sport-related concussion**

Brain Communications, 2026

Richard Wennberg, Audrey Potts, Youssef El-Rahimy, Dominik Zumsteg, Maria Carmela Tartaglia, Charles H. Tator, Paul Comper, Mark Bayley, and Luis Garcia Dominguez

Supplementary Table 1. Patient demographics and results.

Patient	Age, Sex	Acute				1 month				2 months				4 months			
		SCAT	SEF latency (ms)			SCAT	SEF latency (ms)			SCAT	SEF latency (ms)			SCAT	SEF latency (ms)		
Side	Sport	score	iii	vi	vii	score	iii	vi	vii	score	iii	vi	vii	score	iii	vi	vii
1 L	35, F	70	35	89	100	32	36	64	110	4	34	78	94	n/a	n/a		
1 R	Hockey		33	89	98		33	60	105		34	73	94				
2 L	16, M	n/a	36	92	n/d	9	n/a			n/a	n/a			n/a	n/a		
2 R	Hockey		34	93	n/d												
3 L	18, M*	n/a	36	89	n/d	29	34	90	n/d	n/a	n/a			n/a	n/a		
3 R	Hockey		36	128	194		35	125	188								
4 L	23, F	22	33	62	102	0	34	61	110	n/a	n/a			n/a	n/a		
4 R	M-Biking		33	64	108		33	60	101								
5 L	36, F	62	34	56	151	30	35	83	164	18	34	87	163	n/a	34	69	152
5 R	Wrestling		33	70	165		34	66	152		33	69	161		34	66	151
6 L	27, M	51	35	66	112	n/a	n/a			n/a	n/a			n/a	n/a		
6 R	Baseball		35	61	100												
7 L	17, F	94	32	74	n/d	35	33	75	221	25	33	83	113	n/a	32	85	n/d
7 R	F-Hockey		30	58	n/d		31	59	n/d		33	87	145		31	79	n/d
8 L	26, M*	83	39	69	127	6	39	81	136	n/a	40	82	110	n/a	n/a		
8 R	Hockey		39	63	159		38	63	139		n/a (technical)						
9 L	30, M	57	36	76	114	33	33	83	113	n/a	n/a			n/a	n/a		
9 R	Cycling		35	72	113		36	79	104								
10 L	25, M*	79	34	62	90	46	33	67	99	0	34	65	90	0	33	67	90
10 R	Soccer		35	65	77		35	62	106		35	65	92		35	62	80
11 L	31, M	31	32	71	158	15	33	69	n/d	10	32	70	116	1	32	73	141
11 R	Cycling		32	73	140		33	73	126		33	75	113		33	73	119
12 L	29, F*	22	38	86	n/d	n/a	37	64	n/d	n/a	n/a			n/a	n/a		
12 R	F-Football		38	90	n/d		n/a (technical)										
13 L	41, F	93	36	64	134	n/a	n/a			n/a	n/a			n/a	n/a		
13 R	Skating		39	61	108												
14 L	43, F	4	31	57	93	10	31	75	n/d	6	31	70	n/d	3	31	55	97
14 R	Cycling		32	64	n/d		31	70	99		32	66	n/d		32	64	95
15 L	43, M	42	39	123	n/d	0	39	111	152	0	39	99	172	n/a	n/a		
15 R	Basketball		39	108	182		38	105	n/d		38	107	n/d				
16 L	19, M	68	35	101	176	17	34	96	168	0	36	101	n/d	0	35	102	n/d
16 R	Basketball		36	76	129		36	85	113		35	68	116		36	72	131
17 L	31, M	55	37	53	133	28	36	52	119	6	37	61	125	n/a	37	66	123
17 R	Hockey		36	60	115		36	58	109		38	62	116		37	62	126
18 L	24, F	17	n/a (technical)			n/a	n/a			n/a	n/a			n/a	n/a		
18 R	Cycling		37	75	n/d												
19 L	34, F	59	35	79	153	n/a	n/a			20	33	93	163	13	33	85	153
19 R	Skating		31	90	168						31	84	174		33	80	155
20 L	34, M	23	36	84	124	24	37	88	116	n/a	38	84	116	n/a	n/a		
20 R	MMA		39	71	125		38	64	138		39	82	136				
21 L	27, M	62	31	90	133	6	31	94	141	n/a	n/a			n/a	n/a		
21 R	Soccer		31	86	117		31	97	113								

*History of previous sport-related concussions prior to study enrolment: patient 3 (hockey); patient 8 (hockey, football); patient 10 (soccer); patient 12 (soccer, snowboarding).

SCAT = Sport Concussion Assessment Tool 5 (total symptom severity score), SEF = sensory evoked field (latency measured at peak of main component after principal component analysis of magnetoencephalographic data), iii = M30 response, vi = M60 response, vii = M100 response, L = left median nerve stimulation, R = right median nerve stimulation, F = female, M = male, F-Hockey = field hockey, M-Biking = mountain biking, MMA = mixed martial arts, n/d = no definite SEF with M100 topography, n/a = not available. Gray shading = no further patient follow-up.

Supplementary Table 2. Control data.

Control Side	Age, Sex	SEF latency (ms)			
		i	iii	vi	vii
1 L	55, M	23	30	62	108
1 R		22	30	55	108
2 L	19, M	24	34	68	156
2 R		24	35	84	130
3 L	45, M	22	28	52	81
3 R		22	28	50	105
4 L	31, M	24	36	65	87
4 R		23	32	63	85
5 L	30, M	24	36	78	141
5 R		24	33	80	149
6 L	23, M	22	32	76	113
6 R		22	32	76	107
7 L	48, M	26	35	50	n/d
7 R		26	38	63	95
8 L	19, F	22	29	63	115
8 R		21	31	82	n/d
9 L	35, M	23	32	63	130
9 R		23	32	67	104
10 L	17, M	21	35	67	110
10 R		21	31	52	117
11 L	28, F	20	30	63	82
11 R		19	28	62	93
12 L	53, F	21	35	61	n/d
12 R		23	35	68	n/d
13 L	23, F	20	37	52	139
13 R		21	38	60	109
14 L	26, M	22	35	65	130
14 R		22	35	66	130
15 L	15, M	20	33	75	n/d
15 R		19	31	50	n/d
16 L	39, F	24	40	68	119
16 R		22	40	60	113
17 L	40, F	23	33	67	148
17 R		22	33	62	n/d
18 L	31, M	24	39	84	105
18 R		22	29	87	93
19 L	22, F	21	33	77	115
19 R		21	33	71	107
20 L	28, F	21	32	69	113
20 R		20	31	64	108
21 L	33, M	22	35	60	111
21 R		22	35	59	125
22 L	40, M	24	32	51	112
22 R		24	31	50	n/d
23 L	47, M	23	38	65	131
23 R		24	39	62	125
24 L	25, M	22	35	53	122
24 R		22	33	53	125
25 L	24, F	21	33	64	n/d
25 R		21	34	62	99
26 L	24, F	20	31	53	109
26 R		20	31	47	104

SEF = sensory evoked field (measured at peak of main component after principal component analysis of magnetoencephalographic data), i = M20 response, iii = M30 response, vi = M60 response, vii = M100 response, L = left median nerve stimulation, R = right median nerve stimulation, n/d = no definite SEF with M100 topography.

Supplementary Table 3. Area fractional (50%) latency method for M60 peak identification.

Control data.

Control Side	Age, Sex	SEF latency (ms)		
		M60 time range	M60 peak	(L+R)/2 average M60 peak
1 L	55, M	49-91	70	63
1 R		45-68	56.5	
2 L	19, M	50-87	68.5	75
2 R		60-102	81	
3 L	45, M	43-69	56	55
3 R		37-71	54	
4 L	31, M	57-73	65	64
4 R		57-70	63.5	
5 L	30, M	47-102	74.5	75
5 R		47-104	75.5	
6 L	23, M	56-95	75.5	75
6 R		56-94	75	
7 L	48, M	47-75	61	64
7 R		53-79	66	
8 L	19, F	47-73	60	71
8 R		55-109	82	
9 L	35, M	47-82	64.5	65
9 R		52-79	65.5	
10 L	17, M	45-81	63	59
10 R		45-63	54	
11 L	28, F	53-72	62.5	63
11 R		54-72	63	
12 L	53, F	49-68	58.5	64
12 R		50-87	68.5	
13 L	23, F	44-69	56.5	62
13 R		45-89	67	
14 L	26, M	55-96	75.5	78
14 R		47-112	79.5	
15 L	15, M	58-100	79	72
15 R		45-84	64.5	
16 L	39, F	48-94	71	68
16 R		45-83	64	
17 L	40, F	53-93	73	70
17 R		49-84	66.5	
18 L	31, M	47-115	81	81
18 R		54-107	80.5	
19 L	22, F	54-94	74	72
19 R		51-90	70.5	
20 L	28, F	53-96	74.5	72
20 R		47-90	68.5	
21 L	33, M	47-82	64.5	66
21 R		47-88	67.5	
22 L	40, M	42-75	58.5	57
22 R		42-68	55	
23 L	47, M	52-82	67	66
23 R		50-80	65	
24 L	25, M	46-78	62	61
24 R		45-74	59.5	
25 L	24, F	43-96	69.5	67
25 R		43-85	64	
26 L	24, F	37-61	49	53
26 R		37-65	56	

Patient data (acute concussion).

Patient Side	Age, Sex	SEF latency (ms)		
		M60 time range	M60 peak	(L+R)/2 average M60 peak
1 L	35, F	62-132	97	98
1 R		70-127	98.5	
2 L	16, M	54-121	87.5	104
2 R		66-174	120	
3 L	18, M	50-115	82.5	97
3 R		49-172	111	
4 L	23, F	46-92	69	69
4 R		53-85	69	
5 L	36, F	46-98	72	70
5 R		51-85	68	
6 L	27, M	57-80	68.5	67
6 R		46-85	65.5	
7 L	17, F	66-101	83.5	74
7 R		53-74	63.5	
8 L	26, M	48-106	77	76
8 R		45-105	75	
9 L	30, M	61-103	82	85
9 R		52-124	88	
10 L	25, M	56-77	66.5	67
10 R		58-77	67.5	
11 L	31, M	47-117	82	86
11 R		46-132	89	
12 L	29, F	47-129	88	91
12 R		57-132	94.5	
13 L	41, F	49-96	73	70
13 R		49-84	66.5	
14 L	43, F	49-121	85	77
14 R		47-90	68.5	
15 L	43, M	79-151	115	108
15 R		49-151	100	
16 L	19, M	49-150	99.5	88
16 R		50-102	76	
17 L	31, M	44-75	59.5	61
17 R		45-79	62	
18 L	24, F	n/a (technical)		81
18 R		66-96	81	
19 L	34, F	58-132	95	99
19 R		57-146	102	
20 L	34, M	64-105	84.5	79
20 R		47-100	73.5	
21 L	27, M	57-115	86	90
21 R		67-120	93.5	

SEF = sensory evoked field (range = beginning to end of M60 waveform; peak = timepoint dividing range into two equal halves), L = left median nerve stimulation, R = right median nerve stimulation, n/a = not available.

Supplementary Figure 1

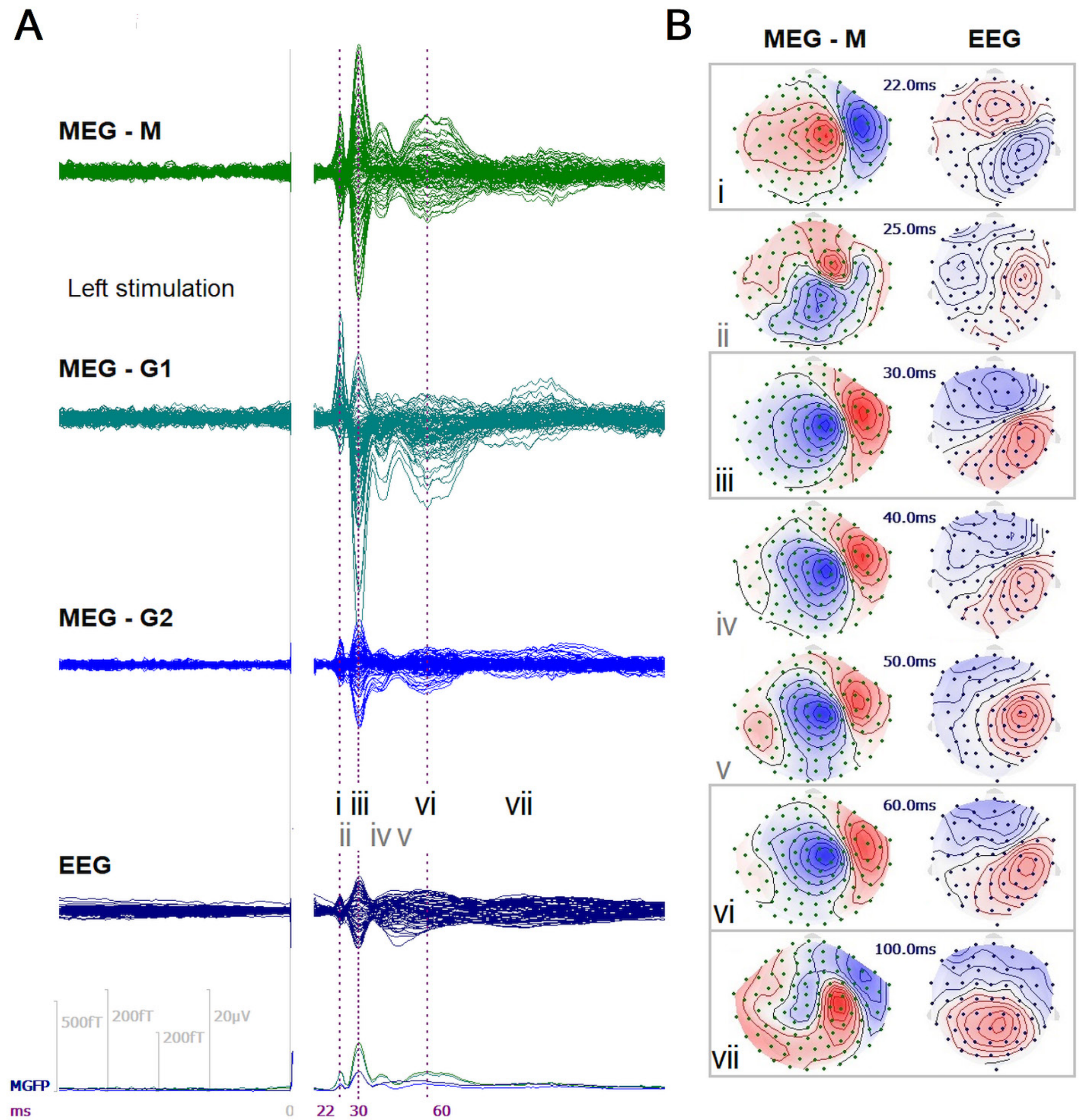

Supplementary Figure 1. Normal somatosensory cortex responses to median nerve stimulation at the wrist. Control subject 1, simultaneous MEG-EEG recording. Left wrist stimulation. (**A**) EEG and MEG evoked responses in primary somatosensory cortex (S1) occur at approximately 20ms (i), 25ms (ii), 30ms (iii), 40ms (iv), 50ms (v) and 60ms (vi); for MEG, the responses at i (M20), iii (M30) and vi (M60) are the main sensory evoked fields (SEFs). The less distinct, lower amplitude evoked response around 100ms (vii) is generated in secondary somatosensory cortex (S2). More details in text and **Supplementary Fig. 2**. Electromagnetic

stimulation artifact (at t=0) removed from figure for clarity. (**B**) Topographic plots show the corresponding MEG magnetometer flux (red = flux out of head, blue = flux into head) and EEG voltage (red = electropositive, blue = electronegative) fields at peaks i-vii. Rectangles highlight the field topographies at the main MEG peaks; by the “right hand rule” of electromagnetism the magnetic flux fields are oriented at 90° to the corresponding voltage fields (see also **Supplementary Fig. 2**). M = magnetometers. G1 = planar gradiometers. G2 = planar gradiometers (orthogonal to G1). MGFP = mean global field power.

Supplementary Figure 2

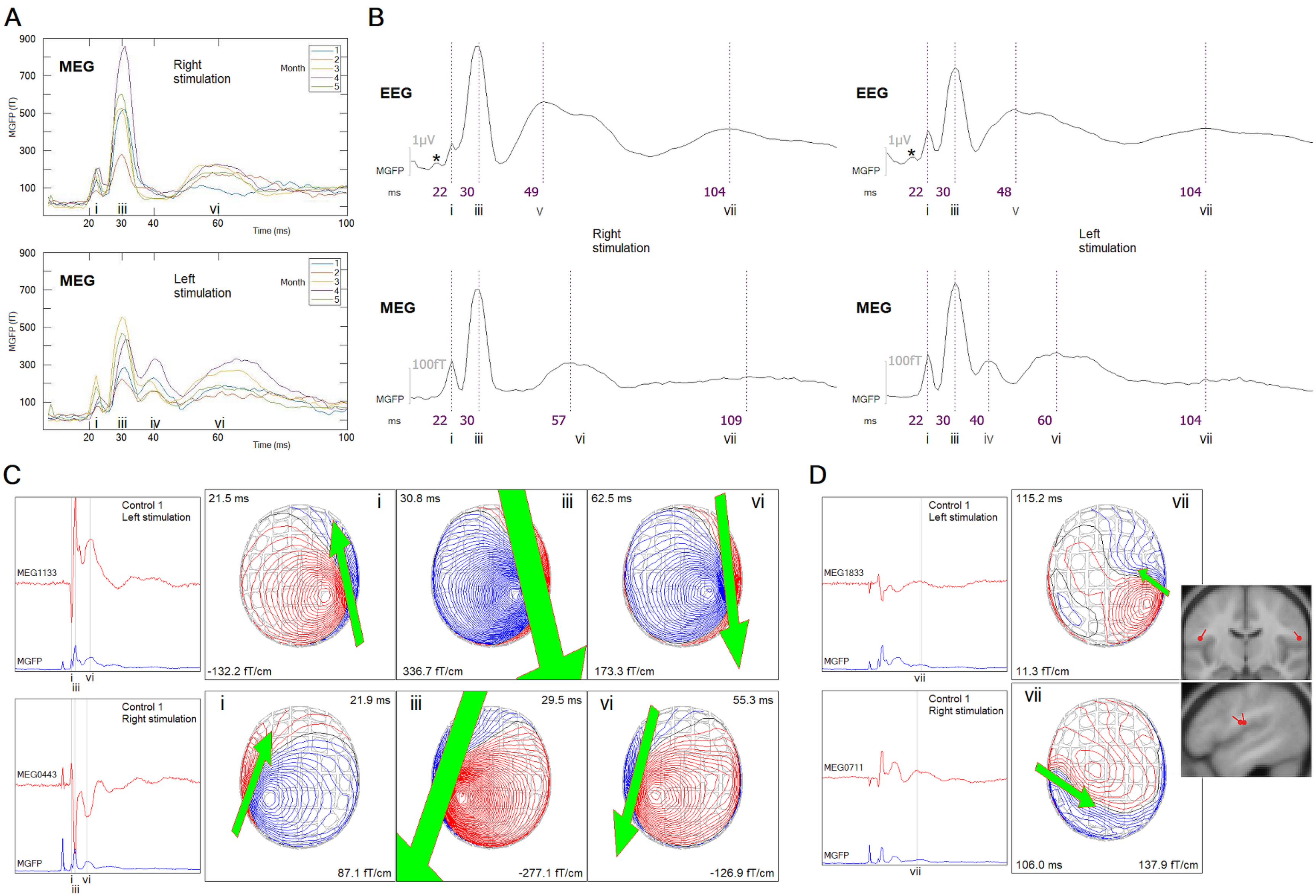

Supplementary Figure 2

Supplementary Figure 2. Replicability and expanded view of EEG and MEG responses to median nerve stimulation at the wrist, topography of flux fields and current flow direction. Control subject 1. (**A**) Consistency of sensory evoked fields (SEFs) recorded in response to right (*top*) and left (*bottom*) median nerve stimulation at the wrist across five MEG recordings acquired at monthly intervals. (**B**) Expanded time scale of the EEG (*top*) and MEG (*bottom*) mean global field power (MGFP) waveforms shown in **Fig. 1** and **Supplementary Fig. 1** (EEG 64 channels, common average reference; MEG 102 magnetometer channels). An early peak at 18ms visible only in EEG (*) is a far-field potential generated subcortically between the upper brainstem and thalamus. Peaks ii and iv, generated in Brodmann area 1, are not separable in the MGFP EEG waveform. Peak iv is visible in the MGFP magnetometer waveform on one side only, presumably due to extension of Brodmann area 1 into the right central sulcus in this subject, producing a tangentially-oriented component of the dipolar field conducive for MEG detection. (**C**) SEFs as recorded from single MEG channels (red) with maximal peak vi (M60) amplitude (*left*) and the flux field topographies (red = flux out of head, blue = flux into head), magnetic field strengths and horizontally-oriented current flow directions (green arrows) corresponding to peaks i (M20), iii (M30 and vi (M60), all generated in Brodmann area 3b (*right*). By the "right hand rule" of electromagnetism the magnetic flux fields are oriented at 90° to the direction of current flow at each peak. (**D**) SEFs as recorded from single MEG channels (red) with maximal peak vii (M100) amplitude (*left*) and the flux field topography, magnetic field strength and vertically-oriented current flow of this later response generated in Brodmann area 43 (*right*), as illustrated by magnetic source imaging (red ball = dipole source maximum; red tail = direction of current flow). Modeling methods as in references[1,2]; forward model = sphere, inverse model = fixed coherent (equivalent current) dipole; solution parameters = goodness-of-fit >70%, signal-to-noise ratio >3.5 and confidence ellipsoids <5.8ml bilaterally; results plotted on the Montreal Neurological Institute averaged brain template.

Supplementary Figure 3

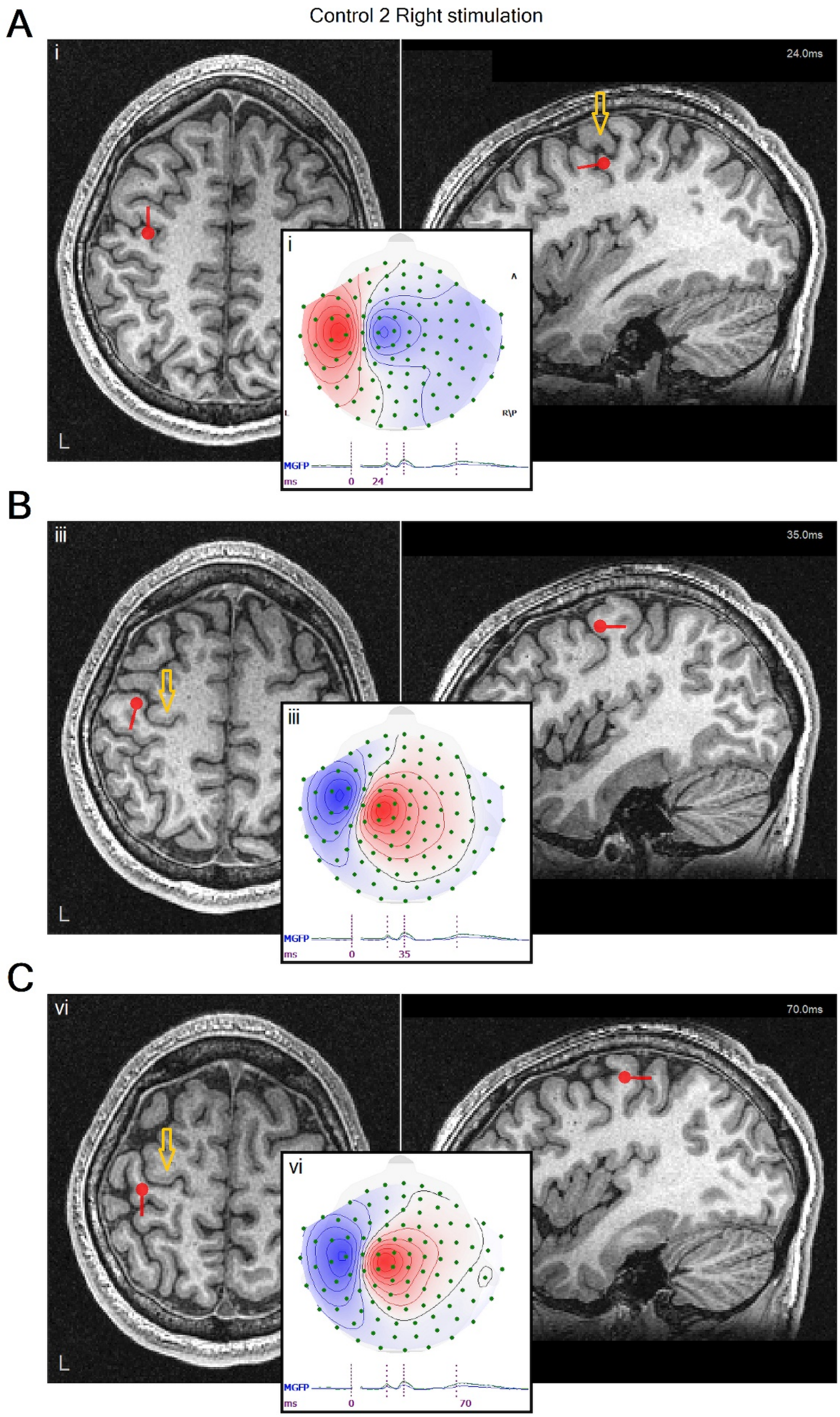

Supplementary Figure 3. Magnetic source imaging localization of the M20, M30 and M60 somatosensory evoked fields. Control subject 2, right median nerve stimulation. Magnetic source imaging of the main S1 somatosensory evoked fields shows (**A**) peak i (M20) generated by anteriorly directed current in the anterior wall of the postcentral gyrus (Brodmann area 3b), (**B**) peak iii (M30) by posteriorly directed current in the same area, and (**C**) peak vi (M60) by posteriorly directed current maximal slightly more superior along the anterior wall of the postcentral gyrus. Red ball = dipole source maximum; red tail = direction of current flow. Arrow = hand area of precentral motor cortex. Modeling methods as in references[1,2]; forward model = sphere, inverse model = fixed coherent (equivalent current) dipole; solution parameters = goodness-of-fit 94.15% (M20), 97.6% (M30), 96.9% (M60), signal-to-noise ratio 4.5 (M20), 11.8 (M30), 10.4 (M60) and confidence ellipsoids 2.0ml (M20), 0.1ml (M30), 0.1ml (M60); results plotted on the subject's brain MRI scan.

Supplementary Figure 4

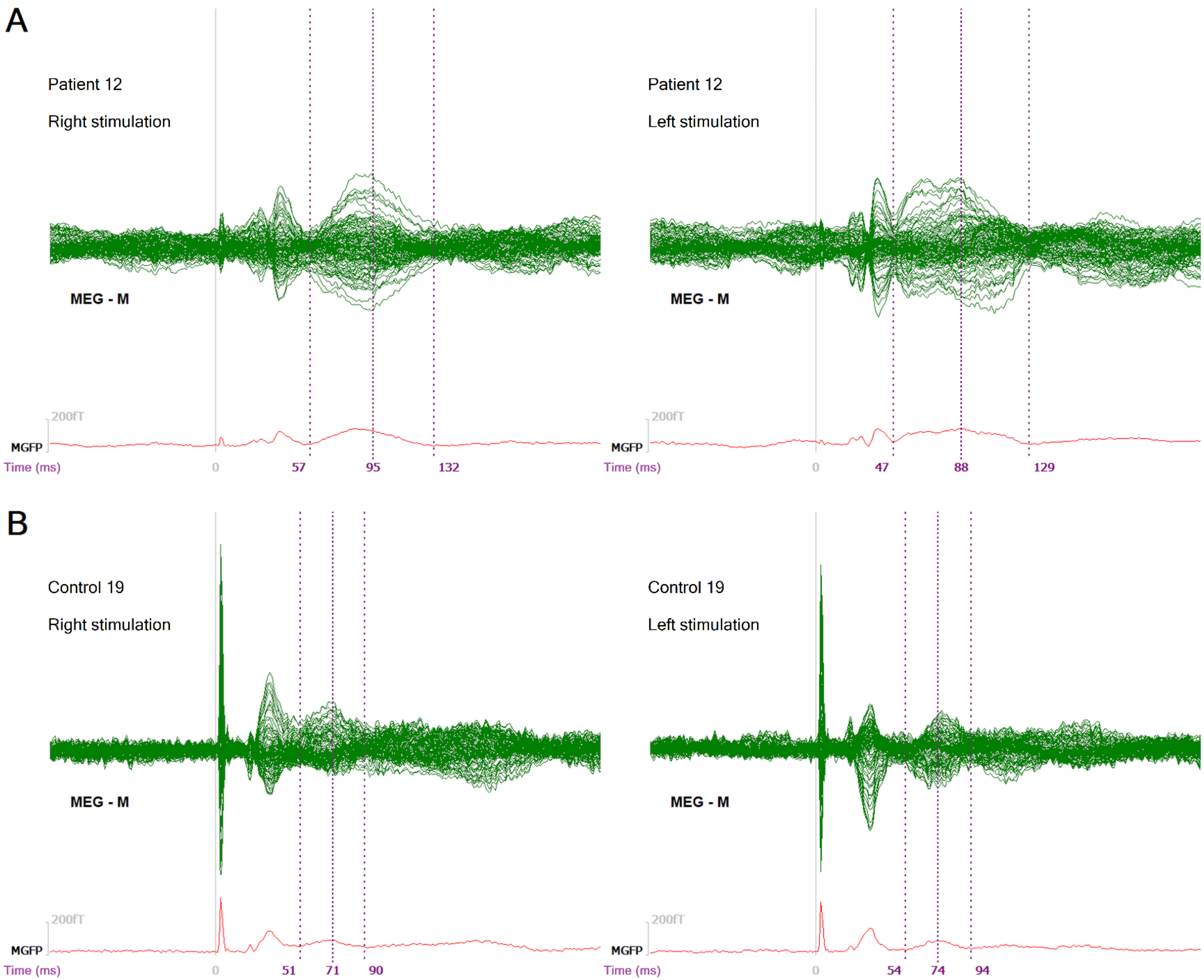

Supplementary Figure 4

Supplementary Figure 4. Area fractional latency method for M60 peak identification. In each panel, left vertical line marks M60 waveform onset and right vertical line marks M60 waveform offset, with the middle line representing the half-way (50%) timepoint, which is used as the "peak" value in this method. (**A**) Patient 12. Temporally dispersed M60 waveforms bilaterally, including separation of the response evoked by left median nerve stimulation into two different amplitude peaks. (**B**) Control 19. Normal bilateral M60 somatosensory evoked fields.

Supplementary Figure 5

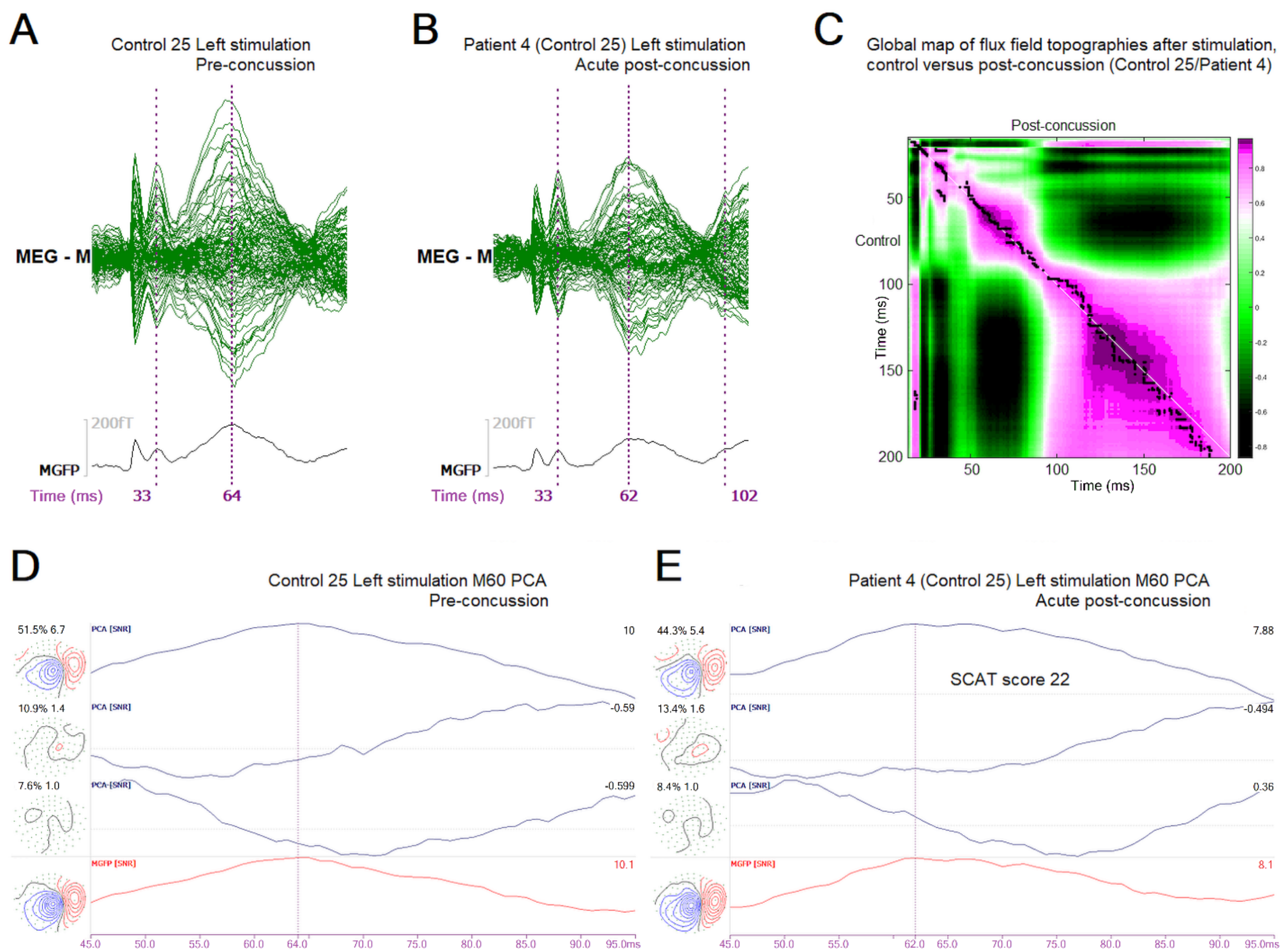

Supplementary Figure 5. Pre-concussion and post-concussion recordings in one individual. Originally control subject 25, this individual later suffered an acute concussion and a repeat recording was acquired, this time as patient 4. The M60 response latencies did not differ between pre-concussion (**A**) and post-concussion (**B**) recordings (right stimulation not shown), and there were no definite flux field topographic dissimilarities between the two recordings (**C**). Colour scale in (**C**) represents normalized topographic similarity, from -1 (low) to +1 (high). Principal component analysis (PCA) across the M60 segment (45ms to 95ms) revealed a peak maximum of the first PCA component at 64ms pre-concussion (**D**) and at 62ms post-concussion (**E**). Upper three PCA rows show the first three extracted components; bottom row shows the mean global field power (MGFP) of the non-decomposed signal. Numbers in the left column of each PCA row show the explained variance (*left*) and signal-to-noise ratio (SNR, *right*) of each extracted component. Numbers at right of each row indicate SNR at timepoint marked by the vertical line. Topographic plots show the magnetometer flux field corresponding to the PCA and MGFP data. SCAT score = Sport Concussion Assessment Tool 5th edition total symptom severity score.

Supplementary Figure 6

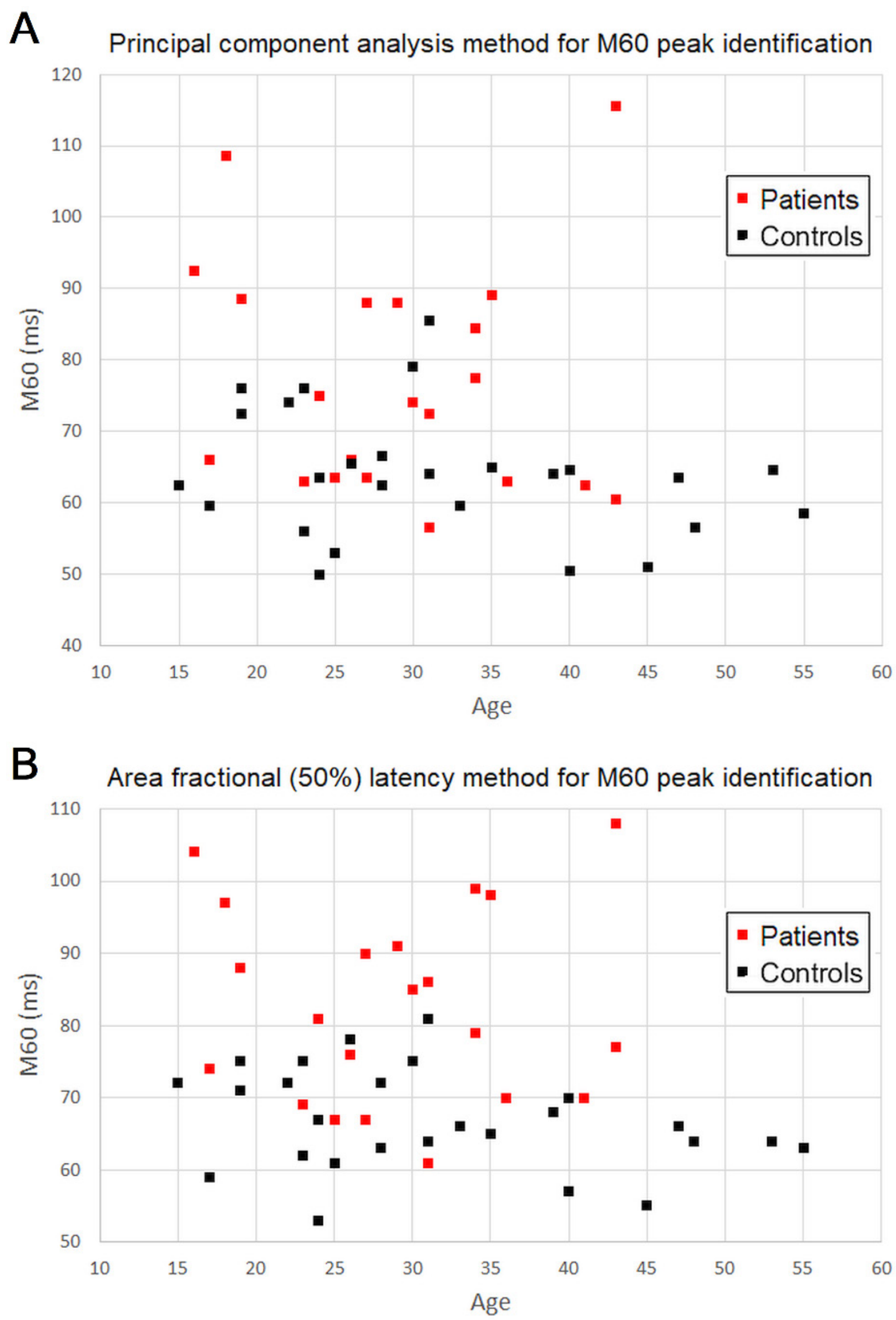

Supplementary Figure 6. Scatterplots of M60 latencies versus age, patients and controls. M60 peak latencies identified by (**A**) principal component analysis (PCA) method or (**B**) 50% area latency method; each datapoint represents the averaged value of the bilateral measurements obtained per subject (except for patient 18, with unilateral measurement). There was no correlation in acute concussion patients between M60 latencies and age (Spearman's rank-order correlation: PCA method, n=21, Spearman's $\rho = 0.022$, $p = 0.93$; 50% method, n=21, Spearman's $\rho = -0.228$, $p = 0.32$).

References

1. Wennberg R, Cheyne D. Reliability of MEG source imaging of anterior temporal spikes: analysis of an intracranially characterized spike focus. *Clin Neurophysiol.* 2014;125:903–918.

2. Wennberg R, Garcia Dominguez L, Del Campo JM. Magnetoencephalographic source localization of the eye area of the motor homunculus. *Can J Neurol Sci.* 2019;46:108-114.
